# Building A National Research Data Management Course for Health Information Professionals

**DOI:** 10.7191/jeslib.2019.1160

**Published:** 2019-07-29

**Authors:** Jessica Van Der Volgen, Shirley Zhao

**Affiliations:** University of Utah, Salt Lake City, UT, USA

**Keywords:** research data management, training

## Abstract

**Background:**

In August 2017 the National Network of Libraries of Medicine Training Office (NTO) was awarded an administrative supplement from the National Library of Medicine (NLM) to create training for librarians in biomedical and health research data management (RDM). The primary goal of the training was to enable information professionals to initiate or enhance RDM at their institutions.

**Case Presentation:**

An eight-week online course was developed to address key concepts in RDM. Each module was organized around measurable learning objectives using existing subject resources, such as readings, tutorials, and videos. Within each module, an expert in the field co-facilitated relevant discussions, created and graded a practical assignment, and answered questions. Thirty-eight participants were selected for this initial cohort. Mentors were assigned to each participant for guidance in completing a required project action plan to further their RDM goals at their institution. The course was evaluated through pre- and post-tests and an online questionnaire.

**Results:**

Thirty participants successfully completed the online course work and project, and gathered at the National Institutes of Health for a Capstone Summit. Students demonstrated improved knowledge of RDM concepts between the pre- and post-tests. Most students also self-reported increased skill and confidence. Practical assignments with individual feedback from experienced data librarians were the most valued aspect of the course. Time to complete each module was underestimated.

**Conclusions:**

The initial offering of this training program improved the RDM skills and knowledge of participants and enabled students to add or enhance services at their institutions. Further investigations are necessary to determine the longer-term impact on the individuals and their libraries. While many of the participants will need additional training to become part of the data-ready workforce of health information professionals, completing this training is an important step in their professional development.

## Introduction and Background

In the Leiter Lecture at the 2017 annual Medical Library Association (MLA) meeting, Dr. Patricia Flatley Brennan, Director of the National Library of Medicine (NLM), shared her vision for the role of NLM in data-powered health. With NLM as a “platform for discovery and pathway for engagement,” medical librarians are critical to this data-intensive era of health care (Leonard 2017). In addition to traditional library skills, such as organization and curation, other skills related to data management and data science will be needed to support health sciences research. Through increased capacity for data management services within health sciences libraries, health information professionals will achieve this vision and directly support the third goal of NLM’s 2017-2027 strategic plan, *A Platform for Biomedical Discovery and Data-Powered Health*, to “build a workforce for data-driven research and health.”

Several professional organizations recognize that specialized skills in data management are now essential for information professionals. The Joint Task Force on Librarians’ Competencies in Support of EResearch and Scholarly Communication, an international group coordinated by the Confederation of Open Access Repositories, published the *Librarians’ Competencies Profile for Research Data Management* in 2016, which includes a detailed list of key knowledge areas such as providing access to data; providing advocacy and support for managing data; and managing data collections. In 2017 MLA released their revised *Competencies for Lifelong Learning and Professional Success* and a self-assessment for its members to appraise their current skill levels in all competencies. Members scored themselves the second lowest among all areas for implementing data management plans, indicating a need for training in competency 2: “a health information professional to be able to curate and make accessible bioscience, clinical, and health information data, information, and knowledge.”

In November 2016 the NTO conducted a national training needs assessment with health sciences information professionals, in which understanding open science concepts and the research data lifecycle were two areas with the largest gaps between self-rated current and desired proficiency. Concurrently, the National Network of Libraries of Medicine (NNLM) formed a Research Data Management Working Group to examine and address the needs of health information professionals who support researchers at their institution in this area. This group conducted an environmental scan focused on what types of data management and data science services were currently being provided by health sciences information professionals. In 188 responses, participants reported services were delivered primarily through web-guides covering research lifecycle tools (REDCap, Vivo, eagle-i, DMPTools, etc.), data visualization tools (R, Python, SAS, SPSS, Tableau, etc.), impact factors, and social media used by researchers (Mendeley, ResearchGate, etc.). The content was a blend of resources authored by library personnel and resources produced outside their library. The questionnaire results indicated that there was little training, consultation, or active support being provided by librarians to researchers. Together, the training needs assessment and environmental scan indicate that health information professionals across the country recognize a need to improve their own skills in these areas. At the minimum, this would translate to having the ability to describe the data lifecycle; identify resources, tools, and repositories; and explain data management or sharing plan requirements of funding agencies.

In the current landscape, training in research data management (RDM) specifically designed for health sciences librarians and information professionals is in its relative infancy compared to the moderately well-established more general RDM online curricula available for professional librarians. Some training materials have been developed with funding from the National Institutes of Health (NIH) Big Data to Knowledge (BD2K) grants and NNLM. For example, the BD2K grants have funded development of courses at Georgetown University, Harvard Medical School, Johns Hopkins University, and New York University (NYU) School of Medicine. NNLM has developed introductory-level courses, including classes on roles for health sciences librarians in healthcare big data and using existing skill sets to support data research. MLA occasionally offers online webinars on related topics, including programming in R, data visualization, precision medicine, and data management diversity. Librarians in the health sciences library community have recently published books on RDM, primarily targeted for librarians new to the topic.

While there are many resources available to learn about data management principles and services, there remains a clear need for a comprehensive training program that brings together the best of these resources and includes meaningful, practical activities focused on biomedical and health research data. In August 2017 the NNLM Training Office (NTO) was awarded an NLM administrative supplement to create that training program specifically for biomedical and health RDM support services. The primary desired outcome of the course was to enable information professionals to add or enhance RDM services at their institutions. The innovative approach to this program was in bringing together novice and experienced data services librarians through mentorship and co-teaching to grow the community of librarians with data management expertise and increase the sustained impact of the program. Guided, applied activities would create a safe place to practice new skills and knowledge in preparation for effecting change. The supplement also specified that the course was to create a cohort of 30 to 40 health information professionals and culminate in a Capstone Summit at the National Institutes of Health.

## Case Presentation

### Developing the Course

NLM recognized that there were already several quality sources for learning RDM and specified in the request for proposals that existing curriculum materials, especially those developed through BD2K, should be used as the primary basis of the training program and minimal effort should be put into creating any new learning materials. Hence, materials were drawn from NYU’s *Research Data Management Training for Information Professionals, The Medical Library Association Guide to Data Management for Librarians*, OHSU’s BD2K OER Modules, *The BD2K Guide to the Fundamentals of Data Science Series*, New England Collaborative Data Management Curriculum Modules, DataONE Education Modules, and Library Carpentry Lessons.

Based on the competencies identified by MLA and the Joint Task Force, the primary course instructor divided key RDM concepts into eight online modules to be delivered on a weekly basis and identified two to five student learning objectives for each module (see Table 1). Foundational or background resources were presented first, followed by current issues and applications. Most modules had a required discussion that either gave some practice in applying the content, asked students to investigate resources at their institution, or required a reflection on the module’s topic. Finally, each module had a practical activity and further resources on the weekly topic. After all assignments were submitted for the week, an answer guide was posted to the module.

In addition to weekly applied assignments, each student was required to complete a capstone project. The project was an action plan for adding or increasing RDM services at their home institutions and was designed to encourage students to use learning from the weekly lessons. The immediate application was intended to reflect the problem-oriented nature of adult learners who desire “to apply tomorrow what [they learn] today” (Knowles 1972, 36). Because of the variety of settings, expertise, strengths, constraints, and goals of the participants, the capstone project was purposely devised to be flexible. In order to keep the project manageable in scope, students were instructed to plan a project that could be completed within four months from the end of the course. Suggested projects included teaching a class, establishing a new service, or conducting a needs assessment or environmental scan. A template with spaces for the project goal, key background information, stakeholders, resources, objectives, timeline, challenges, and proposed solutions was provided. Students were advised to seek advice from their mentors in the design and completion of the project plan.

### Recruiting Experts

To bring a variety of voices and experience to the program, co-teachers, content reviewers, and mentors were recruited through an application process, which included prompts for describing their areas of interest, research, or primary RDM expertise, and for summarizing their qualifications. Announcements were posted to NNLM blogs, the NLM Technical Bulletin, and various relevant listservs. The MLA Data Special Interest Group also helped solicit applicants.

Co-teacher applicants indicated which modules they would like to develop content for and were selected based on their expertise and responses to the prompts. Co-teachers drew on their experiences to create practical, hands-on assignments based on the module objectives. Examples included case studies on making a dataset public, writing a data management plan, determining whether a particular scenario follows FAIR data principles, cleaning a dataset, and crafting an elevator pitch for RDM services. During the course, the co-teachers participated in the module discussions, answered student questions, and provided individual and global feedback on the assignments.

Before the online course opened to students, eight content experts reviewed individual modules for clear, measurable, and appropriate objectives; relevant and engaging content; opportunities for meaningful practice and application of new knowledge; clear instructions and interface usability; and any additional feedback. This feedback was delivered in a written report and used to revise the online class. Modifications included adding clarity to assignment instructions, paring down readings and videos, and addressing other reviewer concerns.

Mentors were selected for their experience and leadership in RDM within libraries, such as research, publications, and education. Each mentor was assigned three-four students in the course. The role of the mentors was to contribute their expertise to course discussion and to advise their mentees on completing their capstone projects. Mentors were expected to guide mentees in the selection and scope of their capstone projects by helping to identify potential obstacles, partners, and stakeholders in at least two meetings with their mentees throughout the course.

### Selecting Students

Students were selected through a separate competitive application process. In addition to contact information, applicants supplied a statement of their experience or interest in RDM, current status of RDM at their institution, and potential benefit for themselves and for their place of employment. A letter of support from the applicant’s supervisor indicating time for participation in the course and commitment to adding or expanding RDM services was required.

**Figure 1:**
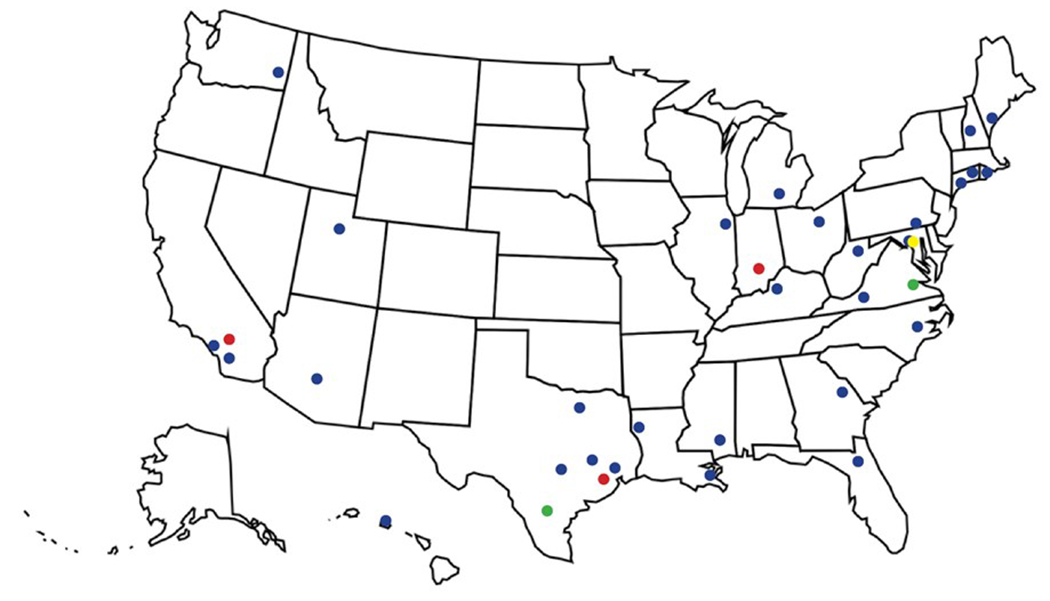
Map depicts locations of participants’ institutions. Academic Libraries are blue, Hospitals are red, Research Institutes are green, and Government Libraries are yellow.

Fifty-four complete applications were received and 35applicants were selected for the initial cohort, along with five alternates. In addition, three NLM staff were selected to join the class. Students represented each of the eight NNLM regions, with the most students from the South Central Region. Students were primarily from academic health sciences libraries, but some came from research institutes, hospital, and government libraries (see Figure 1).

### Running the Program

The online course ran from January to early March 2018. Students completed a pre- and a post-test as part of the evaluation at the end of the online course. The modules opened on a weekly basis and the primary instructor created a short video to address concerns from the previous week and to introduce the current module topic. The co-teacher for each week provided individual feedback to each participant on the assignments, as well as general commentary on any common questions or problems in the assignments.

Between the end of the online course and the Capstone Summit, students worked with their mentors to design a personalized capstone project scoped to be achievable by the end of August 2018. On April 10-11, students, mentors, and staff from NNLM and NLM met at NIH for the Capstone Summit. Participants heard from Dr. Brennan about data science efforts at NLM and from a panel of NIH data scientists. Students presented their projects in small groups and participated in roundtable discussions around key RDM issues, which were moderated by mentors. Members of the NNLM RDM Working Group participated as both facilitators and attendees to learn and plan for the next iteration.

## Results

Thirty-three students completed all of the required components of the online course, including assignments, the pre- and post-tests, capstone project, and a course evaluation. As with any program, course policies were clearly laid out prior to the course but unexpected situations did arise and affected successful participation and completion of the course. Two students withdrew prior to the start of the course due to changing employers; one waitlisted student was offered and accepted the spot. One student was removed from the course after two weeks due to inactivity. Throughout the online course component, three students withdrew from the course due to changing employers or job duty changes (despite institutional commitment). In the end, 30 students attended the in-person Capstone Summit and successfully completed the entire program.

### Evaluation

Evaluations were conducted based on the Kirkpatrick model of training evaluation, in accordance with the submitted proposal. The Kirkpatrick model of training evaluation has long been used by the NTO and is a predominant model for evaluating training across many industries. Level one (participant response) and level two (learning) results are reported here. A level three (behavior change) evaluation was conducted at four months after the conclusion of the online course and results will be reported separately.

At the conclusion of the online course, students (34) completed a 21-item course evaluation. All (100%) students indicated that they learned a new skill that they planned to use in the future. Students reported their expertise had increased from an average of 3.0 (on a scale of 0-10) before the course to an average of 5.4 after the course. Thirty-three (97%) students strongly (25) or somewhat (8) agreed that the course improved their knowledge and skills in research data management, and 33 students strongly (19) or somewhat (14) agreed that the course improved their ability to add or enhance research data management training and services at their institution, which were the primary aims of the course.

Required readings and online modules were ranked by students as the course components that contributed most to their learning, followed closely by assignments and feedback. General discussions ranked lowest. In the comments for this question, the textbook, NYU modules, hands-on assignments with feedback, and perspectives from different instructors were mentioned frequently as “most helpful.” At the time of the evaluation, capstone projects were not complete so students may not have been able to accurately reflect upon its contribution to their learning.

In overall open-ended comments, students indicated the most helpful aspects of the course were the applied assignments, variety of readings and tutorials, and having a broad overview of RDM and related tools. Students also gave specific, critical feedback that can be used to adjust the course to better meet student needs and create a more positive learning experience. The most common criticism by far was that each module took longer than the estimated time. Students indicated that they spent an average of 7.6 hours per week on the course, which was more than the estimate of four hours per week. In particular, several comments indicated that the module on data wrangling was especially challenging and time-consuming. Comments indicated that the assignment was too long and that the preparation for completing it was insufficient. Discussion boards received a mixed reaction. While students enjoyed seeing responses reflecting what was happening at other institutions, the discussions could be too numerous and occasionally too repetitive. Discussions later in the class were broken into smaller groups, and several students indicated that change made it more manageable and useful.

Students were asked how they have used or intend to use what they learned in the course. Many participants indicated plans to initiate services, develop instruction, and share with researchers and colleagues. Below are select comments made about services, consultations, instruction, and sharing.

#### Services & Consultations

“I also am very excited to offer data management consultation services.”

“I would like to use what I learned to actually start data services for the health sciences. The approach to planning and identifying institutional partners was invaluable in that regard.”

“I would like to use the knowledge gained through this course to assist our institution and researchers with research accessibility and reproducibility.”

#### Instruction

“I plan to start RDM services on my campus, most likely in an instructional capacity.”

“I used the resources and information I learned about together in a guide to start teaching about research data management.”

“I will be using materials from this course to develop a research data management workshop and online materials at my institution.”

“I have already started using the information learned in this class by teaching data management classes during Love Data and Endangered Data week.”

#### Sharing

“It is my intent to share my newfound RDM resources, processes, and best practices with researchers (and future researchers, i.e. students) at my institution.”

“I plan to learn more about OpenRefine and I would like to introduce colleagues and faculty to OpenRefine as part of their data management.”

“I will be presenting an overview for my library colleagues at an informal workshop session.”

Several students also indicated early outcomes for themselves or their institutions.

“This course gave me the confidence to “talk the talk” with researchers, administration and other subject librarians.”

“I learned valuable practical knowledge and skills that I can use to create relationships with researchers on my campus.”

“It gave me the chance to explore an area that I have always been interested in. And it provided me with extra motivation to start taking action on starting RDM services on my campus, something I’ve thought about but never started work on until this course.”

“I have a much better understanding of the library roles for each component of the research data cycle.”

### Learning

Knowledge gains were assessed with pre- and post-tests. Both tests consisted of the same 16 questions and were scored out of 10. The pre-test was administered to students prior to the beginning of the online course and the post-test was administered at the conclusion. Thirty-three students successfully completed both the pre-and post-tests. Mean score for the pre-test was 6.21 (median = 6.31, standard deviation = 1.18). Mean score for the post-test results was 7.50 (median = 7.50, standard deviation = 1.13), indicating an average gain of 1.29 (12.9%). Overall, students demonstrated improved knowledge of RDM concepts.

Additionally, capstone projects demonstrated the ability of students to apply what they had learned to their individual settings through action plans for adding or increasing RDM services at their home institutions. The project types focused on Assessment (15), Resource/Tool creation (5), Service enhancement (6), and Training (6). Projects included creating a community of practice, offering instruction on the DMP Tool, conducting needs assessments, working with researchers to make their data available, developing a data institute for librarians, and creating an institutional inventory of datasets.

## Discussion

While very few new resources were created for this class, the organization into topical modules with measurable learning objectives represents a significant contribution to the learning space in biomedical and health RDM. Activities that were created can be modified and reused for subsequent iterations. Offering the course online provided a way to reach students across the country, spread the instruction out over a longer period of time, and allowed students time to investigate institution-specific resources and practices. Many of the applications indicated that their libraries were investigating roles in RDM and did not yet have staff dedicated to RDM services or that the participant was recently hired into this role. The online course provided foundations without significant time away from regular job duties and a way for libraries to develop the skills of their existing staff.

The Capstone Projects required students to examine RDM services in their own contexts and settings, and create an action plan that was suitable for their skills and situation. Developing an action plan gave students a way to demonstrate and apply what they have learned and to provide a path forward for themselves in achieving the goal of adding or enhancing research data management services at their institutions. Student evaluations of the course illustrated that the course and project helped them to gain a better understanding of key stakeholders and partners and their own potential contributions to the status of RDM in their environments.

A follow-up evaluation was conducted in September 2018 to assess how participants have applied their learning to their work and advanced RDM at their institutions. Results are forthcoming, but hopefully the members of the first cohort will continue to learn and grow with each other as they put their action plans into practice, and that lessons learned along the way can be shared back with the growing community of information professionals interested in RDM. To support this effort, the NNLM RDM Working Group has initiated a bi-monthly series of webinars. Topics include, but are not limited to, understanding a library’s role in RDM, getting started, data management planning, and different RDM tools.

In July 2018 NLM awarded NTO a second administrative supplement to improve upon this course, and to develop a new advanced course around data science and open science to increase learning opportunities for these two cohorts and other information professionals ready to advance their knowledge and skills. The goal remains to build a data-ready workforce.

Based on the post-course evaluation, several changes are planned for the next iteration of the RDM course. The estimated time commitment will be increased to five hours per week, and module content will be reduced to better match the weekly time approximation. A course alignment grid will be used to ensure that the objectives are adequately covered without unnecessary repetition. Other changes include moving the “Data Wrangling” module to the more advanced course, distributing the “RDM at Your Institution” content throughout the course, and adding weekly office hours to assist students with assignments, scope their action plans, or answer other questions.

## Conclusion

For many health sciences and information professionals, RDM may not be a current role or part of their job description, but they are trying to expand or alter their role by taking on these duties. Some have been explicitly hired to do so but are new in their role, or it is a new position at their institution. Libraries recognize it as an important new role, but don’t have anyone hired to do so and are trying to expand service areas with current staff. While there are a growing number of training opportunities in RDM and data science from universities, online education programs, and libraries, an organized curriculum specific to biomedical and health sciences research with applied exercises adds significantly to this space.

While the participants may not yet identify themselves as experts in the area, this course did meaningfully improve their knowledge, skills, and ability to add RDM services. Practical activities with individual feedback and a customizable action plan are important components to achieve the goal of enabling students to add or enhance RDM services at their institutions. Subsequent evaluations of this cohort may determine the impact on their organizations and what further training is necessary to include them as part of the data-ready workforce of librarians.

## Figures and Tables

**Table 1:** Learning Objectives by Modules

Week	Module	Objectives
1	RDM Overview and Data Lifecycle	1. Describe how the data lifecycle fits into the larger research lifecycle 2. Articulate the importance of RDM to the research lifecycle 3. Summarize the potential roles of librarians in RDM
2	Data Curation and Documentation	1. Explain what data curation encompasses 2. Explain various types of data (e.g. surveys, video, images) 3. Identify which data elements are important to document 4. Recommend file naming convention based on best practices 5. Check a dataset for potential privacy issues
3	Data Standards, Taxonomies, and Ontologies	1. Distinguish between standards, metadata, taxonomy, and ontology 2. Locate and choose appropriate metadata/descriptors/ontologies for a given dataset 3. Apply selected standards to a given dataset
4	Data Security, Storage, and Preservation	1. Evaluate preservation needs of a dataset (e.g. file format, software) 2. Identify appropriate data repositories for a given dataset 3. Discuss potential solutions for datasets with security/privacy issues (HIPAA) 4. Explain how policies affect data ownership, security, and storage
5	Data Sharing and Publishing	1. Articulate the FAIR data principles 2. Explain the importance of research reproducibility 3. Describe the concept of “open data” and challenges for sharing biomedical research data 4. Explain data sharing incentives, data citations, and data journals
6	Data Management Plans	1. Explain DMP requirements of funding agencies (NIH, NSF) 2. Create a DMP that meets the requirements of a selected funding agency
7	Data Wrangling	1. Articulate how RDM supports research reproducibility and data science 2. Implement best practices for creating data files 3. Use OpenRefine to clean data
8	RDM at Your Institution	1. Identify key stakeholders in RDM at your institution 2. Practice advocating and supporting RDM
